# Targets for parathyroid hormone in secondary hyperparathyroidism: is a “one-size-fits-all” approach appropriate? A prospective incident cohort study

**DOI:** 10.1186/1471-2369-15-132

**Published:** 2014-08-13

**Authors:** Emmanuelle Laurain, Carole Ayav, Marie-Line Erpelding, Michèle Kessler, Serge Briançon, Laurent Brunaud, Luc Frimat

**Affiliations:** 1 Department of Nephrology, University Hospital, University of Lorraine, Vandœuvre-lès-Nancy, France; 2 EA 4360 Apemac, Nancy University, P. Verlaine Metz University, and Paris Descartes University, Nancy, France; 3 Department of General, Digestive and Endocrine Surgery, University Hospital, University of Lorraine, Vandœuvre-lès-Nancy, France

**Keywords:** Dialysis, Secondary hyperparathyroidism, Cinacalcet, Pharmacoepidemiological study

## Abstract

**Background:**

Recommendations for secondary hyperparathyroidism (SHPT) consider that a “one-size-fits-all” target enables efficacy of care. In routine clinical practice, SHPT continues to pose diagnosis and treatment challenges. One hypothesis that could explain these difficulties is that dialysis population with SHPT is not homogeneous.

**Methods:**

EPHEYL is a prospective, multicenter, pharmacoepidemiological study including chronic dialysis patients (≥3 months) with newly SHPT diagnosis, i.e. parathyroid hormone (PTH) ≥500 ng/L for the first time, or initiation of cinacalcet, or parathyroidectomy. Multiple correspondence analysis and ascendant hierarchical clustering on clinico-biological (symptoms, PTH, plasma phosphorus and alkaline phosphatase) and treatment of SHPT (cinacalcet, vitamin D, calcium, or calcium-free calcic phosphate binder) were performed to identify distinct phenotypes.

**Results:**

305 patients (261 with incident PTH ≥ 500 ng/L; 44 with cinacalcet initiation) were included. Their mean age was 67 ± 15 years, and 60% were men, 92% on hemodialysis and 8% on peritoneal dialysis. Four subgroups of SHPT patients were identified: 1/ “intermediate” phenotype with hyperphosphatemia without hypocalcemia (n = 113); 2/ younger patients with severe comorbidities, hyperphosphatemia and hypocalcemia, despite SHPT multiple medical treatments, suggesting poor adherence (n = 73); 3/ elderly patients with few cardiovascular comorbidities, controlled phospho-calcium balance, higher PTH, and few treatments (n = 75); 4/ patients who initiated cinacalcet (n = 43). The quality criterion of the model had a cut-off of 14 (>2), suggesting a relevant classification.

**Conclusion:**

In real life, dialysis patients with newly diagnosed SHPT constitute a very heterogeneous population. A “one-size-fits-all” target approach is probably not appropriate. Therapeutic management needs to be adjusted to the 4 different phenotypes.

## Background

In the 70’s, secondary hyperparathyroidism (SHPT) was described as a severe bone disease occurring in young end-stage renal disease (ESRD) patients with significant duration of dialysis. When parathyroid hormone (PTH) was very high, up to 1000 ng/L, associated with hypercalcemia, the only treatment was subtotal parathyroïdectomy [1,2]. In the 90’s, access to kidney transplantation for the young and dialysis for the old led to a rapid ageing of dialyzed population [3]. During the first decade of the millennium, a new paradigm emerged. First, SHPT was considered not only as a bone disease, but also as a vascular disease [4]. Second, SHPT turned out to be a biological rather than a clinical disease at bedside [5]. A “one-size-fits-all” approach was recommended: PTH under 300ng/L from 2003 to 2009 according to K-DOQI [6]. Due to variability in PTH measurement, the target was modified in 2009: “maintaining PTH levels in the range of approximately two to nine times the upper normal limit for the assay” [7,8]. Third, cinacalcet tends to be seen by the clinicians as the most appropriate solution for the treatment of SHPT due to its mechanism of action, when conventional therapy is not effective enough [6,9]. But the randomized controlled EVOLVE study, published in 2012, failed to demonstrate efficacy of cinacalcet to reduce the risk of death or major cardiovascular events [10]. Today, the exact importance of PTH is still debated [11].

Large observational cross-sectional studies about SHPT have recently been published [12-15]. An incidence/prevalence bias may have hampered a precise description of SHPT phenotypes [16]. In order to capture the phenotypes of SHPT at bedside, we meticulously enrolled all dialysis patients of the REIN registry - Region of Lorraine with newly marked PTH elevation in a prospective observational study from December 2009 to May 2012. At inclusion, we delivered a validated questionnaire to measure clinical symptoms. With an original statistical analysis, we demonstrated that high PTH levels matched with 4 very different phenotype profiles, suggesting that a “one-size-fits-all” target approach for SHPT was not appropriate.

## Methods

The pharmacoepidemiological EPHEYL (Étude PHarmacoÉpidémiologique de l’hYperparathyroïdie secondaire en Lorraine) study is a 2-year, open-cohort, prospective, observational study on incident SHPT, i.e. newly diagnosed, with a 2-year follow-up, set in the 12 dialysis units located in the French region of Lorraine (public or private).

### Inclusion criteria

Adult patients included in EPHEYL were on dialysis (hemodialysis or peritoneal dialysis) for at least 3 months with one of the following criteria: for the first time 1) PTH ≥ 500ng/L, 2) initiation of cinacalcet, 3) parathyroidectomy if severe SHPT. The PTH cut-off value of 500 ng/L was chosen at the time of 2003 K-DOQI [6]. Indeed, when we initiated the study, the updated KDIGO recommendations were not effective, and PTH levels between 150 and 300 ng/L were advocated [6,8]. The indication for parathyroidectomy or the use of a calcimimetic were retained when PTH level was ≥ 500 ng/L, hence the choice of this threshold in our study.

From 1^st^ December, 2009 to 31^st^ May, 2012, all patients who were on dialysis for at least 3 months were identified through the REIN registry - Region of Lorraine [17]. The occurrence of one out of the three inclusion criteria was prospectively followed up in all these patients.

Patients were included in the study at the time of PTH dosing, initiation of cinacalcet, or parathyroidectomy.

Physicians were encouraged to adhere to the KDIGO™ guidelines updated in 2009 [8].

### Data collection

The following socio-demographic and clinical data were retrieved from the REIN registry: age, gender, body mass index (BMI), type of dialysis, dialysis vintage, primary etiology of nephropathy, comorbidities (smoking, diabetes, cardiovascular diseases, hypertension, respiratory diseases and cancer), and being on renal transplant waiting list [17]. The vast majority of patients were Caucasians.

BMI was described as a continuous quantitative variable and obesity (BMI >30 kg/m^2^) as a binary variable. Primary etiology of nephropathy was classified into diabetic nephropathy, vascular nephropathy, glomerulonephritis, pyelonephritis, hereditary nephropathy, and other/unknown. Cardiovascular diseases comprised history of heart failure, cardiac heart disease, acute coronary syndrome, arrhythmia, peripheral arterial disease, and stroke. Respiratory diseases encompassed history of chronic respiratory insufficiency, asthma, and obstructive pulmonary disease. Hypertension was considered present if: diastolic and/or systolic blood pressure greater than 80 and 130 mm Hg, respectively, or a current antihypertensive therapy.

SHPT symptoms experienced by patients were assessed by using: 1) the Parathyroidectomy Assessment of Symptoms (PAS) questionnaire. This self-administered questionnaire validated in dialysis patients with SHPT assessed the severity of 14 SHPT symptoms (Table 1) experienced by patients using a visual analog scale (VAS) which ranged from 0 (not experiencing any symptom) to 100 (experiencing the most extreme aspect of the symptom) [18-20]. This questionnaire was administered at the time of inclusion. PAS scores were analyzed as quantitative variables or proportion of patients with at least one symptom scoring more than 0; 2) the collection of clinical signs reported in medical records such as osteoarticular pain, myasthenia, bone fractures, paresthesia, pruritus, tetany, and calciphylaxis. A patient was symptomatic if at least one symptom had a PAS score greater than 0 or was reported in his medical records.

**Table 1:** Specific symptoms assessed by the parathyroidectomy assessment of symptoms (PAS) questionnaire, a self-administered disease-specific outcome tool, in patients with secondary hyperparathyroidism (SHPT)

	**Items in PAS questionnaire**
1.	Pain in the bones
2.	Feeling tired easily
3.	Mood swings
4.	Feeling “blue” or depressed
5.	Pain in the abdomen
6.	Feeling weak
7.	Feeling irritable
8.	Pain in the joints
9.	Forgetfulness
10.	Difficulty to stand up
11.	Headaches
12.	Dry skin
13.	Being thirsty
14.	Pruritus

The following biological parameters were collected at the time of inclusion: PTH, calcemia, phosphorus, vitamin D, alkaline phosphatase (ALP), albumin, hemoglobin and measured ionized calcium. KDIGO™ guidelines have recommended to maintain PTH up to 2 to 9-fold above the normal range [8]. PTH was therefore described as binary variable (in or out of target) and quantitative variable (multiple of upper normal limit). According to KDIGO™ guidelines, calcemia was checked as hypo-, normo- or hypercalcemia using 2.1 and 2.6 mmol/L as cut-off values; phosphatemia as hypo-, normo- et hyperphosphatemia using 0.8 and 1,45 mmol/L as cut-off values. ALP was analyzed according to 2 stages using medians as cut-off values, albuminuria according to 2 stages using 25 g/L as cut-off value, and hemoglobin according to 3 stages using 10 and 12 g/dL as cut-off values.

Four technologies were used for PTH dosing: chemiluminometric technology (48%), electro cheminulometric method (23%), immuno-enzymology (11%), immune chemiluminometric technology (16%), and unknown (2%); each kit was provided by several laboratories which had different standards.

All drugs acting on phospho-calcic metabolism were collected and classified into 4 groups: vitamin D and analogs, calcium supplementation, calcium-free phosphorus binders, and cinacalcet.

A standardized form was used to collect data from medical records. A Steering Committee consisting of an epidemiologist (CLA) and a nephrologist (LF) reviewed all forms and medical records when collected biological data were out of international standards.

### Ethics statement

This study was conducted in compliance with French regulations concerning pharmaco-epidemiological studies [21]. Approvals from the French data protection agency (CNIL: n° 904163) and from the Advisory Committee on information processing research in the field of health located in the region of Lorraine (CCTIRS: n° 0428) were obtained through the national REIN registry. An information sheet was displayed in all dialysis units, and each patient was given an individual written information sheet at the initiation of dialysis.

### Statistical analyses

Patient characteristics were described as proportions for categorical variables, and means and standard deviations (SD) for continuous variables, except PAS scores described as medians.

Multivariate analyses using multiple correspondence analysis (MCA) and ascendant hierarchical clustering on clinical, biological and therapeutic characteristics of SHPT were performed to identify subgroups of patients [22]. All these parameters were binary variables.

MCA was applied to determine the major axes summarizing more clearly data [22]. This method gives a set of coordinates of the categories of variables, and thus reveals the relationships between the individuals and the different categories. Each principal component was interpreted in terms of amount of contribution for each category to variance of axis. The contribution of a variable was statistically significant when its mean was greater than 1/p, (p = number of categories of variables). Graphical evaluation was built using the major components in a series of two-dimensional graphs.

Then an ascendant hierarchical clustering was used to determine the number of subgroups on the basis of coordinates of the main components retained by MCA. The 4 clustered subgroups were numbered according to the order of the selection for the classification. The validity of this method was measured by the “cubic clustering criterion” with a cut-off of 2. After subgroups were selected, χ^2^ tests were performed to compare and highlight parameters defining each distinct profile of patients.

The statistical analyses were performed using SAS software, version 9.2 (SAS Institute, North Carolina, US).

## Results

### Patients

**Figure 1:**
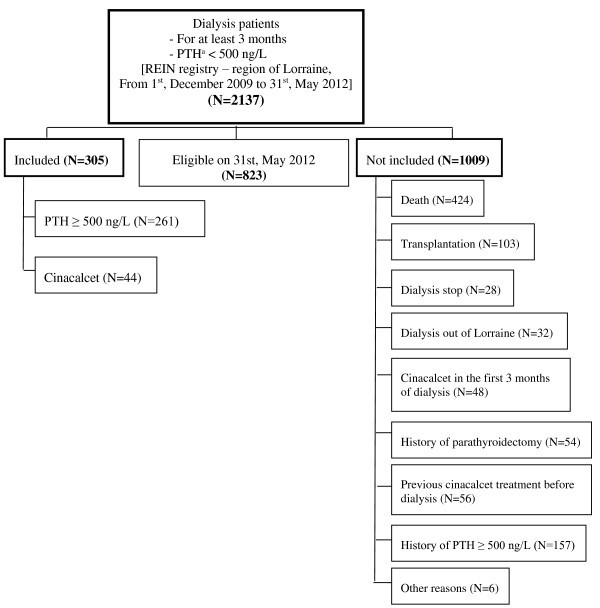
**Disposition of patients. **^a^Parathyroid hormone.

A total of 2137 patients who were on dialysis for at least 3 months, with PTH < 500 ng/L were retrieved from the REIN registry – Region of Lorraine between 1^st^ December, 2009 and 31^st^ May, 2012. Among them, 305 patients were included in the EPHEYL study: 86% with an incident PTH ≥500 ng/L (n = 261), 14% with an initiation of cinacalcet (n = 44), and 0% with a first-line parathyroidectomy (Figure 1).

**Figure 2:**
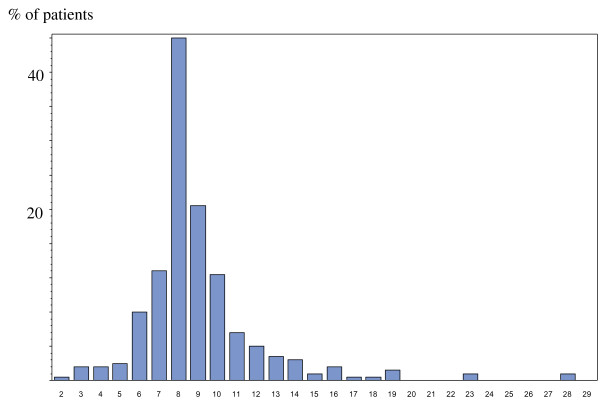
Distribution of parathyroid hormone (PTH) according to multiples of the upper normal limit for the assays in the EPHEYL study.

There was no statistically significant difference in socio-demographic and clinical characteristics between both groups according to inclusion criteria (Table 2). Regarding PTH levels, 10 different values for the upper normal limit were obtained, and ranged from 38.8 to 638 ng/L (median value: 560 ng/L). Despite these high values PTH remained in the KDIGO™ target range in 64% of patients.The distribution of PTH according to multiples of the upper normal limit revealed that 60% of patients maintained PTH up to 8-fold above the upper normal limit (Figure 2).

**Table 2:** Socio-demographic, clinical and biological characteristics for the EPHEYL population according to inclusion criteria

			**Inclusion criteria**				
	**Study population**		**Cinacalcet**		**PTH**^**a**^ **≥ 500 ng/L**		
	**(N = 305)**		**(N = 44, 14.4%)**		**(N = 261, 85.6%)**		
**Variables**	**N**		**N**		**N**		***p***
Age^b^, years	305	66.6 ± 15.3	44	63.3 ± 14.2	261	67.1 ± 15.4	*0.122*
Gender (males), %	305	60	44	71	261	58	*0.126*
BMI^c,b^, kg/m^2^	301	28.6 ± 6.9	44	28.3 ± 6.3	257	28.7 ± 7.1	*0.697*
>30 kg/m^2^, %	301	33.8	44	29.5	257	34.5	*0.522*
Type of dialysis, %	304		44		261		*0.340*
Hemodialysis		91.8		95.5		91.2	
Peritoneal dialysis		8.2		4.5		8.8	
Dialysis vintage^b^, months	305	27.5 ± 34.5	44	23.1 ± 24.5	261	28.3 ± 35.9	*0.362*
On transplant waiting list, %	300		44		256		*0.636*
Yes		8.3		11.4		7.9	
No		91.7		88.6		92.1	
Primary etiology of nephropathy, %	300		43		257		*0.211*
Diabetic nephropathy		16.0		23.3		14.8	
Vascular nephropathy		16.3		20.9		15.6	
Glomerulonephritis		10		11.6		9.7	
Pyelonephritis		4.7		7.0		4.3	
Hereditary nephropathy		7.7		11.6		7.0	
Others/Unknown		45.3		36.2		48.7	
Comorbidities, %	305		44		261		
Cardiovascular diseases		49.5		45.5		50.2	*0.561*
Diabetes		44.9		31.8		47.1	*0.590*
Respiratory diseases		11.5		15.9		10.7	*0.319*
Cancer		13.1		6.8		14.2	*0.181*
Hypertension		85.9		88.6		85.4	*0.573*
Smoking		13.4		13.6		13.4	*0.675*
Clinical signs, %	305		44		261		
Reported in medical records		27.2		31.8		26.4	*0.458*
Reporded in PAS^d^ questionnaire		44.9		31.8		47.1	*0.059*
*Pain in the bones*^*e*^	125	17	12	6	110	21	*0.317*
*Feeling tired easily*^*e*^	131	47	13	50	115	46	*0.054*
*Mood swings*^*e*^	124	23	13	20	108	23	*0.216*
*Feeling “blue” or depressed*^*e*^	123	6	12	6	108	6	*0.184*
*Pain in the abdomen*^*e*^	124	12	11	4	110	13	*0.055*
*Feeling weak*^*e*^	126	48	12	23	111	49	*0.067*
*Feeling irritable*^*e*^	123	20	12	21	108	20	*0.081*
*Pain in the joints*^*e*^	128	40	13	52	112	39	*0.130*
*Forgetfulness*^*e*^	126	12	14	12	109	12	*0.317*
*Difficulty to stand up*^*e*^	121	18	10	6	108	20	*0.020*
*Headache*^*e*^	115	5	11	13	101	5	*0.372*
*Dry skin*^*e*^	122	35	13	49	106	30	*0.325*
*Being thirsty*^*e*^	121	51	13	51	105	51	*0.265*
*Pruritus*^*e*^	122	20	12	49	107	16	*0.302*
Symptomatic patients, %	122	58	12	52.3	107	59	*0.402*
Biological parameters							
PTH^a,b^, ng/L	305	619 ± 226	44	468 ± 262	261	644 ± 210	*<0.0001*
*within KDIGO target*^*f*^*, %*	305	64.3	44	81.8	261	61.3	*0.009*
Serum calcium^b^, mg/L	305	87.0 ± 7.1	44	81.8 ± 7.1	261	87.1 ± 7.2	*0.681*
Calcemia, %	305		44				*0.935*
*≤ 84 mg/L*		42.6		43.2		42.5	
*84-104 mg/L*		57.4		56.8		57.5	
*> 104 mg/L*		0		0	261	0	
Serum phosphorus^b^, mg/L	305	54.4 ± 16.5	44	50.9 ± 17.6	261	55.0 ± 16.3	*0.129*
Phosphatemia, %	305		44				*0.675*
*<25 mg/L*		2.6		2.3		2.7	
*25-45 mg/L*		28.6		34.1		27.6	
*>45 mg/L*		68.9		63.6		69.7	
Serum alkaline phosphatase^b^, UI/L	299	103.4 ± 56.9	43	124.3 ± 97.0	256	99.9 ± 46.3	*0.009*
Bicarbonatemia^b^, mmol/L	303	22.2 ± 3.4	44	22.4 ± 2.8	258	22.1 ± 3.5	*0.618*
Albumin^b^, g/L	291	36.3 ± 4.6	42	36.7 ± 4.1	249	36.2 ± 4.6	*0.504*
Hemoglobin^b^, g/dL	279	10.3 ± 1.6	44	10.6 ± 1.5	235	10.3 ± 1.6	*0.245*
Vitamin D^b^, ng/mL	209	19.2 ± 13.5	31	15.8 ± 10.4	178	19.7 ± 13.9	*0.138*
Ionised calcium^b^, mmol/L	58	1.1 ± 0.2	3	0.9 ± 0.8	55	1.1 ± 0.1	*0.031*
Treatment, %	305		44		261		
Vitamin D (per os)		63.9		72.7		62.5	*0.189*
Calcium		68.9		81.8		66.7	*0.045*
Phosphorus binders		59.3		61.4		59 0	*0.768*
Cinacalcet at inclusion		14.4		100		0	*<0.0001*
Cinacalcet at 3 months		27.2		56.8		22.2	*<0.0001*

^a^Parathyroid hormone; ^b^Results are presented as mean ± SD; ^c^Body mass index; ^d^Parathyroidectomy Assessment of Symptoms; ^e^Median value; ^f^The 2009 updated KDIGO^TM^ (Kidney Disease: Improving Global Outcomes) guidelines have recommended target range for serum PTH to 2–9 times upper the normal range [8].

Among the 44 patients treated with cinacalcet, 36 patients had PTH within the KDIGO™ target range before initiating the treatment. At 3 months of inclusion, 19 patients (43%) discontinued treatment with cinacalcet.

### Cluster analysis

**Figure 3:**
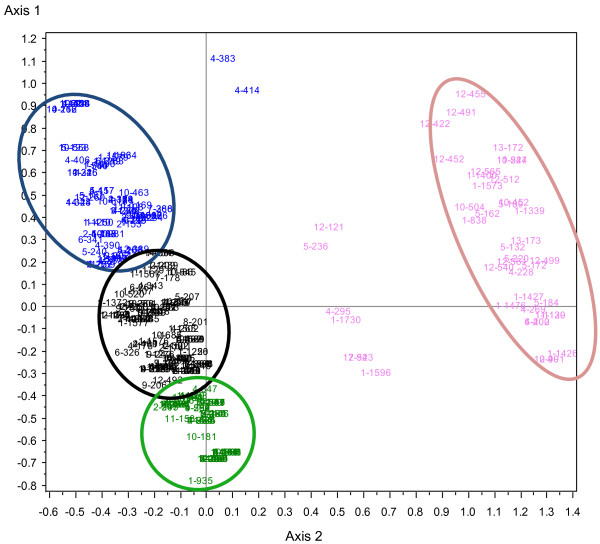
**Identification of four distinct subgroups of dialysis patients with secondary hyperparathyroidism (SHPT) using multiple correspondence analyses.** The horizontal axis defined the presence or absence of calcium supplementation, the presence or absence of treatment with cinacalcet, and serum PTH below or above 500 ng/L. The vertical axis defined a normophosphatemia, a hyperphosphatemia, the absence or presence of phosphorus binders, high or low level of alkaline phosphatases, the presence or absence of vitamin D supplementation, the presence or absence of calcium suplementation. Each patient is identified by a number and a color according to the following code: black for group 1 (“intermediate”), green for group 2 (younger with severe cardiovascular comorbidities), blue for group 3 (elderly patients with few cardiovascular comorbidities), pink for group 4 (“cinacalcet prescription”).

Ascendant hierarchical clustering identified four subgroups of patients according to their SHPT profiles as shown Figure 3. The “cubic clustering criterion” of the model was 14, higher than the cut-off of 2, validating the classification.

The four clustered subgroups of patients were named according to their main characteristics regarding variables used (Table 3) or not used for clustering patients (Table 4):

**Table 3:** Characteristics of dialysis subgroups identified at time of secondary hyperparathyroidism (SHPT) diagnosis: variables used to cluster dialysis patients at time of SHPT diagnosis

**Variables**	**Group 1**^**a**^	**Group 2**^**a**^	**Group 3**^**a**^	**Group 4**^**a**^	
	**(N = 113, 37.2%)**	**(N = 73, 24.0%)**	**(N = 75, 24.7%)**	**(N = 43, 14.1%)**	***p***
Clinical signs, %					
All clinical signs	64	55	56	54	*0.512*
Clinical signs reported in PAS^b^	55	45	37	33	*0.031*
Biological parameters					
PTH^c,d^, ng/L	666 ± 182	630 ± 199	635 ± 233	449 ± 255	*<0.0001*
*within KDIGO target, %*	51	66	69	86	*0.0004*
Serum calcium^d^, mg/L	89 ± 7	82 ± 6	89 ± 6	87 ± 7	*<0.0001*
*Hypocalcemia, %*	29	81	27	42	*<0.0001*
Serum phosphorus^d^, mg/L	58 ± 17	62 ± 13	44 ± 12	52 ± 17	*<0.0001*
*Hyperphosphatemia, %*	81	96	25	67	*<0.0001*
*Hypophosphatemia, %*	4	4	0	2	*<0.0001*
Serum alkaline phosphatase^d^, UI/L	100 ± 44	78 ± 29	120 ± 53	123 ± 99	*<0.0001*
*Under median value, %*	50	84	21	53	*<0.0001*
Treatments, %					
Vitamin D and analogs	73	84	24	77	*<0.0001*
Calcium supplementation	68	95	35	86	*<0.0001*
Calcium-free phosphorus binders	58	93	25	65	*<0.0001*
Cinacalcet	0	0	3	98	*<0.0001*

^a^Group 1: “intermediate” patients, Group 2: younger patients with severe comorbidities, Group 3: elderly patients with few cardiovascular comorbidities, Group 4: patients who initiated cinacalcet; ^b^Parathyroidectomy Assessment of Symptoms questionnaire; ^c^Parathyroid hormone; ^d^Results are presented as mean ± SD; *p* corresponds to ANOVA.

**Table 4:** Characteristics of dialysis subgroups identified at time of secondary hyperparathyroidism (SHPT) diagnosis: variables not used to cluster dialysis patients at time of SHPT diagnosis

**Variables**	**Group 1**^**a**^	**Group 2**^**a**^	**Group 3**^**a**^	**Group 4**^**a**^	
	**(N = 113, 37.2%)**	**(N = 73, 24%)**	**(N = 75, 24.7%)**	**(N = 43, 14.1%)**	***p***
Socio-demographic					
Age^b^, years	65 ± 17	66 ± 14	72 ± 13	63 ± 15	*0.008*
Gender (males), %	56	66	55	70	*0.217*
Dialysis					
Type of dialysis, %					*0.330*
*Hemodialysis*	90	96	91	95	
*Peritoneal dialysis*	10	4	9	5	
Dialysis vintage^b^, months	27 ± 32	19 ± 22	38 ± 48	24 ± 24	*0.007*
Comorbidities, %					
Cardiovascular diseases	48	62	41	47	*0.086*
Diabetes	46	55	41	30	*0.070*
BMI^c^, kg/m^2^	29	30	27	28	*0.217*
*>30 kg/m*^*2*^*, %*	37	42	23	30	*0.091*
Biological parameters					
Albumin^b^, g/L	37 ± 5	36 ± 5	36 ± 5	37 ± 4	*0.396*
Hemoglobin^b^, g/dL	10.0 ± 1.6	10.4 ± 1.5	10.6 ± 1.7	10.6 ± 1.5	*0.046*

^a^Group 1: “intermediate” patients, Group 2: younger patients with severe comorbidities, Group 3: elderly patients with few cardiovascular co-morbidities, Group 4: patients who initiated cinacalcet; ^b^Results are presented as mean ± SD; ^c^Body mass index; *p* corresponds to ANOVA.

– “Intermediate” patients (Group 1, 37%): patients with hyperphosphatemia without hypocalcemia, sharing similar characteristics with the next group of patients (Group 2) but better controlled

– Younger patients with severe cardiovascular comorbidities (Group 2, 24%): most often obese or diabetic patients, with shorter dialysis vintage, mainly with hyperphosphatemia and hypocalcemia despite multiple medical treatments, suggesting poor adherence

– Elderly patients with a few cardiovascular comorbidities (Group 3, 25%): rarely obese, with longer dialysis vintage, mainly with normophosphatemia and normocalcemia despite few patients with SHPT treatment, with health status appearing to be, at first, much better than the one in group 2

– Patients who initiated cinacalcet (Group 4, 14%): 42 out of the 44 patients who initiated cinacalcet and another patient were classified into a clearly distinct subgroup (Figure 3). Two patients treated with cinacalcet were classified into other groups.

## Discussion

EPHEYL is a well-characterized cohort of patients with incident severe SHPT diagnosis described not only on the basis of initiation of cinacalcet but also a cut-off value for PTH [6]. An incident population helps to accurately describe diseases, avoiding bias related to incidence and prevalence mix [16]. We used an appropriate methodology, MCA and ascendant hierarchical clustering, to identify homogeneous subgroups of cases with a high statistical level validity [22]. Our four clustered subgroups consisted of homogeneous patients with same medical history, same prior therapy, and probably similar characteristics concerning mineral bone diseases and cardiovascular co-morbidities.

SHPT symptoms are difficult to assess due to the lack of specificity. The self-administered questionnaire developed by Pasieka *et al.* was used in several studies on primary and secondary hyperparathyroidism to quantify severity of symptoms using median values [18-20]. In the EPHEYL study, one out of two patients suffered from at least one symptom. But the most frequent symptoms (thirst, weakness, fatigue, and pain of joints) were not specific. As the questionnaire was developed in the context of parathyroidectomy, its validity is questionable at early stage of SHPT.

The PTH cut-off value of 500 ng/L was chosen at the time of 2003 K-DOQI [6]. Its enabled to focus on SHPT patients without adynamic bone disease [8,23]. Furthermore, no patient had hypercalcemia, suggesting that there was no tertiary or autonomized SHPT. This result is consistent with the incident type of our cohort, as tertiary SHPT were found in previous studies including prevalent SHPT patients [6,24]. Nevertheless, we know that PTH is subject to many simultaneous types of variability [7,11]. Our study points out obstacles with the use of PTH to precisely diagnose SHPT. The distribution of PTH at a cut-off value of 500, according to the new recommendation: “maintaining PTH levels in the range of approximately two to nine times the upper normal limit for the assay” was wide (Figure 2). Jean *et al.* have suggested that PTH should be replaced with specific biochemical markers of bone such as bone ALP and beta cross-laps to follow-up SHPT [24]. These measurements, however, are too costly to be recommended in routine clinical practice [8]. Finally, in the context of quite vague recommendations, clinicians should be aware that a binary approach for SHPT diagnosis, i.e. absence/presence, is not adequate. There is definitely a grey zone for diagnosis which limits are not easily defined. We should recommend an observation period before acting strongly.

In this grey zone, our study identified four statistically distinct subgroups of patients. Our description of each group reflected a clinical reality, and was therefore clinically appropriate. Noteworthy, at bedside, these distinct phenotypes should be distinguished by doctor rather by biological cut-offs. This pleads for patient-doctor contact. A recent publication has demonstrated a positive association between patient-doctor contact and outcomes [25]. Last but not least, our study reinforces the recent publication by Levin that has recommended acknowledging the heterogeneity of chronic kidney disease populations and appropriately characterizing populations for studies [26].

The group of “elderly patients with a few cardiovascular comorbidities”, in majority with normocalcemia and normophosphatemia, had a PTH which, at first, should impressed clinicians. In another hand, normal serum phosphorus could not be explained by malnutrition; despite their old age, nutritional markers (such as albumin and phosphatemia) were not statistically different from those in the other groups. PTH seemed to be associated with a good clinical condition and a low prevalence of comorbidities. These results are consistent with those from previous studies in showing that, particularly in elderly, PTH is inversely correlated with score of comorbidities [12,27]. At the time of diagnosis of SHPT, this subgroup of patients did not require presumably an intensive therapeutic management.

The group of “younger patients with severe cardiovascular comorbidities”, by contrast, consisted of a majority of patients (66%) with PTH within targets [8]. However, the mean PTH was similar to the one found in the previous group. They seemed to be most likely to have bone and cardiovascular complications, as in previous cohorts, possibly linked to hyperphosphatemia, with a high proportion of cardiovascular diseases, diabetes and obesity [4,13]. As a result, they should require an intensive care management. Of note, most of them had hyperphosphatemia and hypocalcemia despite multiple treatments. It is obvious that this phenotype is characterized by very low adherence to first-line strategies for SHPT, e.g. calcium, vitamin D and phosphorus binders. For this treatment category, a recent meta-analysis has pointed out poor adherence in a majority of patients [28].

The group of “intermediate patients” seemed to show intermediate characteristics between those with both previous groups. Most of them had hyperphosphatemia without hypocalcemia and the highest PTH. They seemed to be more likely to be at high risk for cardiovascular events due to uncontrolled hyperphosphatemia; as a result, they should be cautiously monitored [21].Finally, the group of patients “who initiated cinacalcet” was very different from the others (Figure 3). Most of patients had hypocalcemia and hyperphosphatemia. Patients were given already multiple conventional treatments before cinacalcet initiation. At the time of cinacalcet initiation, an overwhelming majority of patients (86%) had PTH within KDIGO™ targets which were applicable during the study period. Our study has confirmed that cinacalcet was prescribed for broadened indications in real life, and highlighted that the benefit-risk balance of cinacalcet was not favorable in patients with low PTH. This phenotype “cinacalcet user” including such patients should not exist. Before such therapeutic agents were marketed, indications for surgical parathyroidectomy were limited to symptomatic SHPT patients with very high PTH (>1000 ng/L). Initially presented as an alternative to parathyroidectomy, cinacalcet is now prescribed in pre-symptomatic patients with PTH > 300 ng/L, perhaps due to previous KDOQI recommendations.

Its prescription is based on studies suggesting that cinacalcet should have a protective effect on cardiovascular disease outcomes and reduce risk of fractures [29]. Recently, the prospective EVOLVE study failed to demonstrate the role of cinacalcet in reducing cardiovascular mortality and events [10]. These negative results can be explained, in particular, by the fact that 23% of patients in the treated group and 11% in the placebo group received the trade formulation of cinacalcet, which skewed the results. The EPHEYL study has shown that the use of cinacalcet was likely to be physician-dependent rather than characteristic of patients, and decision-making to prescribe it was insufficiently detailed. Thus in the group of patients who initiated cinacalcet, 43% of them who should have been treated with cinacalcet were not taking treatment 3 months later. Considering that low PTH levels characterize adynamic bone disease and/or a high prevalence of comorbidities, we should currently recommend to prescribe cinacalcet only after conducting a rigorous investigation on its benefit/risks [23,27]. The strength of our study was to identify a subgroup of incident SHPT patients treated with cinacalcet despite low PTH in a real life setting. The matter whether cinacalcet should be contraindicated in patients with low PTH is of great interest.

Our study has some limitations. Its observational nature which may seem like a drawback is strength actually. Pharmacoepidemiological study, as EPHEYL, reflects routine clinical practices with various therapeutic strategies which are not always consistent with previous randomized trials [10]. Second, we have taken into account only data at the inclusion, making sense as regards to the incident nature of a disease. The 2-year follow-up of the EPHEYL cohort should reinforce the relevance of the classification. Third, most of patients were included with a PTH cut-off value of 500ng/L, while the latest KDIGO™ guidelines have recommended maintaining PTH in ranges, at approximately 2 to 9 times the upper normal limit, rather than absolute values. KDIGO™ recommendations were not implemented when we wrote our article. As a result, data on PTH have been detailed in this report. Fourth, the methodology allowed identification of distinct profiles, not completely disjunctive. It is therefore possible that some patients of distinct subgroups should have partially similar characteristics at junctions. Notwithstanding differences between groups are relevant. Fifth, clinical symptoms assessed by PAS questionnaire need to be interpreted with caution, due to the difficulty met to collect data from self-administered questionnaire.

## Conclusion

In conclusion, four significantly distinct profiles of dialysis patients with a recent severe SHPT diagnosis were identified on the basis of clinical, biological and therapeutic data routinely available. Our well-characterized incident cohort, coupled with an original methodological approach, highlights a contemporary picture of daily clinical practice. Our study reinforces that the benefit-risk balance of cinacalcet is not positive in patients with low PTH, and raises the matter whether cinacalcet should be contraindicated in such patients. A “one-size-fits-all” target for PTH approach is probably not appropriate. Therapeutic management needs to be adjusted to the four different phenotypes.

## Competing interests

The authors declare that they have no competing interests.

## Authors’ contributions

EL conceived the study, participated in its design, analysis and interpretation of data, and drafted the manuscript. CA was involved in the design of the study, acquisition, analysis and interpretation of data, and was involved in the statistical analysis. MLE participated in the design of the study and performed the statistical analysis. MK was involved in the design of the study and interpretation of the data. SB was accountable for all aspects of the analysis of data. LB was involved in the design of the study and interpretation of the data. LF participated in the general supervision of the work, helped to draft the manuscript, and revised it critically for important intellectual content. All authors read and approved the final manuscript.

## Pre-publication history

The pre-publication history for this paper can be accessed here:

http://www.biomedcentral.com/1471-2369/15/132/prepub
